# The Antibacterial Activity of Australian *Leptospermum* Honey Correlates with Methylglyoxal Levels

**DOI:** 10.1371/journal.pone.0167780

**Published:** 2016-12-28

**Authors:** Nural N. Cokcetin, Matthew Pappalardo, Leona T. Campbell, Peter Brooks, Dee A. Carter, Shona E. Blair, Elizabeth J. Harry

**Affiliations:** 1 The ithree institute, University of Technology Sydney, Sydney, NSW, Australia; 2 University of the Sunshine Coast, Maroochydore, QLD, Australia; 3 University of Sydney, Sydney, NSW, Australia; Universiti Sains Malaysia, MALAYSIA

## Abstract

Most commercially available therapeutic honey is derived from flowering *Leptospermum scoparium* (manuka) plants from New Zealand. Australia has more than 80 *Leptospermum* species, and limited research to date has found at least some produce honey with high non-peroxide antibacterial activity (NPA) similar to New Zealand manuka, suggesting Australia may have a ready supply of medical-grade honey. The activity of manuka honey is largely due to the presence of methylglyoxal (MGO), which is produced non-enzymatically from dihydroxyacetone (DHA) present in manuka nectar. The aims of the current study were to chemically quantify the compounds contributing to antibacterial activity in a collection of Australian *Leptospermum* honeys, to assess the relationship between MGO and NPA in these samples, and to determine whether NPA changes during honey storage. Eighty different *Leptospermum* honey samples were analysed, and therapeutically useful NPA was seen in samples derived from species including *L*. *liversidgei* and *L*. *polygalifolium*. Exceptionally high levels of up to 1100 mg/kg MGO were present in *L*. *polygalifolium* honey samples sourced from the Northern Rivers region in NSW and Byfield, QLD, with considerable diversity among samples. There was a strong positive relationship between NPA and MGO concentration, and DHA was present in all of the active honey samples, indicating a potential for ongoing conversion to MGO. NPA was stable, with most samples showing little change following seven years of storage in the dark at 4°C. This study demonstrates the potential for Australian *Leptospermum* honey as a wound care product, and argues for an extension of this analysis to other *Leptospermum* species.

## Introduction

Honey has been used therapeutically by many cultures for millennia and has re-emerged as a topical treatment for wound and skin infections, mainly due to its antimicrobial activity [1]. In particular, honey produced from the *Leptospermum scoparium* (manuka) plant from New Zealand exhibits broad-spectrum antimicrobial activity against a diverse range of bacterial and yeast pathogens, and is equally effective against multi-drug resistant bacteria [2–6]. Resistance to the antimicrobial effects of manuka honey on growing bacterial cells has not been reported nor has it been induced under laboratory conditions [2, 3]. There are a number of medicinal honey products on the market that use manuka honey from New Zealand, or the Australian equivalent produced from other *Leptospermum* species. However, despite being registered with medical regulatory authorities as wound care agents in Australia, Canada, the European Union, Hong Kong, New Zealand and the USA, their use in mainstream medicine remains limited [7], largely due to a lack of understanding of all of the mechanisms by which *Leptospermum* honey kills pathogens. The growing crisis of antibiotic resistance [8] has revived interest in the clinical use of honey, particularly in wound care and its efficacy against drug-resistant pathogens.

Several properties of honey contribute to its antimicrobial activity. In most honeys, it is the high osmolarity, low pH, and, most predominantly, the enzymatic production of hydrogen peroxide that exerts an antimicrobial effect [9]. The level of antimicrobial activity is highly variable and depends on the floral source, processing and storage conditions of the honey, prevailing environmental conditions that affect the physiology of the floral species, and bee-related factors such as age or colony health [10–16].

Antibacterial activity that remains in honey following neutralisation of hydrogen peroxide is commonly referred to as “non-peroxide activity” (NPA). NPA is equivalent to the “Unique Manuka Factor” (UMF®), a trademark registered by the UMF Honey Association and available for use under licence by producers of manuka honey from New Zealand. NPA was first identified in manuka honey from New Zealand and is closely associated with the floral source as it is generally derived from phytochemical components produced by manuka and related *Leptospermum* species [10, 17], although a small number of non-*Leptospermum* honeys have also shown this type of activity [10, 18–21]. Honey with high NPA is highly sought after for use in medical-grade honey products as it is not destroyed by the catalase present in body fluids, and can withstand gamma irradiation [22], which is used for sterilisation. The compound primarily responsible for the NPA of manuka honey has been identified as methylglyoxal (MGO), which forms from the nectar-derived compound, dihydroxyacetone (DHA), during the ripening of honey [23–25]. Consequently, the level of MGO in honey is dictated by factors including the DHA content of the nectar, the extent of *Leptospermum* bloom and the foraging behaviour of honey bees [25, 26].

There are a number of microbiological and chemical tests that are used in the honey industry to determine the suitability of honey as an antibacterial product. Antibacterial activity is generally determined by an agar well diffusion method, which measures the extent to which *Staphylococcus aureus* is inhibited with reference to a phenol standard [10]. NPA is often marketed in New Zealand honey as the UMF, with a rating >10+ considered therapeutically active. DHA, which converts to MGO during storage, is an indicator of the potential for honey to increase in activity. DHA and MGO levels in honey can be directly assessed by high-performance liquid chromatography (HPLC) [26, 27]. MGO levels can then be used to estimate the NPA, where a honey with MGO >260 mg/kg is predicted to have >10+ activity based on the relationship between MGO and NPA identified previously [23]. Finally, hydroxymethylfurfural (HMF), which occurs spontaneously in all honey types, is often quantified by HPLC as a measure of honey freshness (and as a measure of spoilage) as it increases in concentration with storage length and temperature. Levels of HMF in honey above 40mg/kg are undesirable as they can mean honey producers will not be able to sell or export the honey.

The wealth of evidence that supports New Zealand manuka honey as a modern healthcare option has translated to commercial success for the industry. However, New Zealand will not be able to support the likely growing demand for medical-grade honey [28]. Furthermore, New Zealand honey supply is at risk due to infection of their honey bee (*Apis mellifera*) populations by the parasitic varroa mite [29], which is associated with colony collapse disorder, an alarming phenomenon that significantly threatens global honey production [30]. Australia is home to 83 of the 87 known *Leptospermum* species [31, 32] (including *L*. *scoparium* [25]) and is still free of the varroa mite, however very few studies have investigated the properties of honey derived from Australian *Leptospermum* honey [2, 12, 27, 33]. In a comprehensive survey of Australian honey for antibacterial activity, Irish *et al*. reported high levels of NPA in some Australian *Leptospermum* honeys [12], however this work pre-dated the discovery of MGO as the major antibacterial component of *Leptospermum* honey, and DHA, MGO and HMF levels in these honey samples were not ascertained at the time.

The aim of the current study was to revisit the 80 Australian *Leptospermum* honeys collected by Irish *et al*. [4] and to determine their levels of DHA, MGO and HMF as well as their current antibacterial activity (NPA). This study is the first to investigate the relationship between the NPA of Australian *Leptospermum* honey and its phytochemical constituents, and the change in this activity over time. We report a strong positive correlation between MGO content and NPA in these honeys and also demonstrate that the non-peroxide activity is preserved for up to seven years after original harvest when honeys are stored in the dark at 4°C.

## Materials and Methods

### Honey samples

Eighty honeys derived from *Leptospermum* species were selected from the 477 Australian honey samples collected by Irish *et al*. [12] between March 2005 and June 2007 (S1 Table). These samples had been catalogued, stored in sterile specimen jars and refrigerated in the dark at 4°C at the University of Sydney, Australia, since collection. A database of information pertaining to these samples was compiled by Irish *et al*. [12] that included collection date, geographic region and floral source. Identification of the floral source of the honey was performed at the time of collection by the apiarists based on the local floral species in bloom prior to honey harvesting, location of the apiary and organoleptic characteristics of the honey. The majority (70) of the 80 Australian *Leptospermum* honey samples were received from the Northern Rivers region of NSW, with the remaining collected from various regions across the country (S1 Fig). All honey samples were identified as being from four monofloral sources (defined as >50% nectar from one floral source [34]) and four multifloral sources. Multifloral and monofloral *L*. *polygalifolium* honeys from Northern Rivers comprised 55% of the samples in this collection.

A sample of unprocessed manuka honey and two samples of commercially available Medihoney^TM^, provided by Comvita New Zealand Ltd., were used as positive controls. Tasmanian clover honey (with no detectable DHA or MGO) was used as a negative control for chemical analysis, and was supplied by the University of the Sunshine Coast, Australia. The control honey samples were also stored at 4°C and in the dark.

### Quantitation of phytochemical components in Australian *Leptospermum* honey samples

Chemical analyses of honey samples were performed using methods adapted from Windsor *et al*. [27, 35]. Briefly, this method involved accurately weighing 0.30–0.40 g of honeys in duplicate into 75 mm test tubes. Hydroxyacetone internal standard (0.250 ml, 0.528 mg/ml) was weighed into the test tubes and the entire sample dissolved in 1.2 ml of O-(2,3,4,5,6-pentafluorobenzyl) hydroxylamine (PFBHA) reagent (0.2 g per 10 ml 0.1 M citrate buffer at pH 4). Derivatisation of MGO, DHA and 5-hydroxymethylfurfural (HMF) with PFBHA took 75 minutes. The samples were diluted with water and acetonitrile until homogeneous, and quantified in duplicate by Reverse Phase-High Performance Liquid Chromatography (RP-HPLC; Perkin Elmer^TM^ Series 200 HPLC and Autosampler, Binary Pump and Flexar® Photodiode Array Detector with a ZORBAC Eclipse Plus column (75 mm x 4.6 mm, 3.5 μm) utilising a 75:25 water: acetonitrile to 100% acetonitrile mobile phase program).

MGO, DHA, HMF (Sigma-Aldrich, St Louis, MO, USA) standards were used for six point calibration, and hydroxyacetone (Sigma-Aldrich) was used as the internal standard for quantitation. All standard solutions were stored at -20°C. Quantitation standards were prepared by dissolving known concentrations of the standard solutions in six aliquots of clover honey (which has no detectable MGO, DHA or HMF unless spiked).

#### Chemical profile data analysis

Data collection and peak area integration were completed with Chromera® Chromatography Data Systems for Liquid Chromatography. The calibration curves were derived from the quantitation standards by plotting the masses of MGO, DHA and HMF in each of the spiked clover honey standards against their peak area ratios relative to the hydroxyacetone internal standard. The concentration of each compound was determined as mg/kg of honey. Descriptive statistical analysis (i.e. means, standard deviation, range and medians) was conducted using IBM® SPSS® Statistics Version 20 (Armonk, NY, USA).

### Determining the non-peroxide antibacterial activity of Australian *Leptospermum* honey samples

Non-peroxide antibacterial activity (NPA) of honey samples with reference to phenol were determined using methods reported in Irish *et al*. [12]. *Staphylococcus aureus* ATCC 25923 (Oxoid, Hampshire, UK) was used as the test organism for the phenol assays. Honey solutions were freshly prepared in a 50% weight per volume (w/v) solution in sterile deionised water, and further diluted to 25% volume per volume (v/v) in a 2 mg/ml catalase solution (3187 units/mg; Sigma-Aldrich) to remove any hydrogen peroxide activity.

Medihoney^TM^ (Comvita Ltd., New Zealand) with predetermined UMF® (>15) and clover honey (with no detectable NPA) were both prepared with catalase as above, and were included in each assay as a positive and negative control, respectively. Phenol (Sigma-Aldrich) standards of 2%, 3%, 4%, 5%, 6%, and 7% v/v were prepared daily in sterile deionised water.

Aliquots (100 μl) of each honey sample and phenol solutions were dispensed into the wells of the assay plates, in duplicate. Quasi-latin squares were used to randomise the sample distribution and plates were incubated at 37°C for 18–24 h.

The diameter of each zone of inhibition was measured at perpendicular angles using digital Vernier callipers (accurate to 0.1 mm; Kinechrome Australia Pty Ltd.). The mean diameter of the zone of inhibition was calculated and squared, and a standard curve was generated using the phenol standards (concentration of phenol versus mean squared diameter). The NPA of honey was calculated using the standard curve, multiplied by 4.69 to account for the dilution and density of honey (based on a mean honey density of 1.35 mg/ml [36]), and expressed as the equivalent phenol concentration (% v/v, subsequently referred to as %). Each honey sample was tested in duplicate on three separate occasions.

#### Antibacterial activity data analysis

All data were analysed qualitatively and statistical analysis of change in activity over time was performed with Minitab Express (v.1.3.0) statistical software (State College, PA, USA), using the Wilcoxon signed ranks test. The correlation between NPA and MGO was determined using the Spearman’s Rank Correlation test performed with R (v. 3.3.1) statistical software (Vienna, Austria).

## Results

### Quantitation of DHA, MGO and HMF in Australian *Leptospermum* honey samples

Monofloral *L*. *polygalifolium* honeys sourced from Northern Rivers (n = 29) and Byfield in QLD (n = 1) exhibited the highest levels of both MGO and DHA in the study as shown in Table 1 and S1 Table. Most of these samples (n = 21) possessed MGO levels of >500 mg/kg, and DHA levels of >2000 mg/kg, with marked variation from one sample to another. Six additional honeys sourced from Northern Rivers had high levels of MGO including a monofloral *L*. *liversidgei* honey (704 mg/kg), a multifloral *L*. *liversidgei* honey (418 mg/kg), an *L*. *laevigatum* honey (583 mg/kg) and three multifloral *L*. *semibaccatum* samples (407–430mg/kg). The DHA content for these six honeys ranged considerably (79–2541 mg/kg).

**Table 1:** Methylglyoxal (MGO) and dihydroxyacetone (DHA) levels in Australian *Leptospermum* honey samples.

*Leptospermum* species	Region^a^	No. of samples tested	MGO^b^ (mg/kg)		DHA^c^ (mg/kg)	
			Range	Mean ± SD	Range	Mean ± SD
*L*. *continentale*	Central VIC	2	222–262	242 ± 28	775–1092	934 ± 224
*L*. *laevigatum*	Central VIC	1	NA	14 ± 0	NA	0 ± 0
	Northern Rivers NSW	1	NA	583 ± 9	NA	1485 ± 33
Multifloral *L*. *laevigatum*	Hunter NSW	1	NA	10 ± 5	NA	0 ± 0
	Northern Rivers NSW	1	NA	5 ± 1	NA	12 ± 17
*L*. *liversidgei*	Northern Rivers NSW	5	208–704	361 ± 205	226–882	454 ± 282
Multifloral *L*. *liversidgei*	Northern Rivers NSW	13	8–362	113 ± 127	61–818	246 ± 231
*L*. *polygalifolium*	Byfield QLD	1	NA	1100 ± 57	NA	2182 ± 150
	Northern Rivers NSW	28	146–1118	628 ± 243	157–3425	2042 ± 971
Multifloral *L*. *polygalifolium*	Northern Rivers NSW	12	0–526	313 ± 149	0–1262	689 ± 459
*L*. *semibaccatum* multifloral	Northern Rivers NSW	4	407–430	419 ± 10	427–736	628 ± 138
Unspecified *Leptospermum* sp.	Murraylands SA	1	NA	0 ± 0	NA	0 ± 0
	Northern Rivers NSW	6	418–836	519 ± 158	206–1705	1205 ± 556
	Stradbroke Island QLD	1	NA	835 ± 36	NA	2343 ± 131
	Unknown	3	126–995	560 ± 5	79–501	255 ± 219

^a^NSW: New South Wales, QLD: Queensland, SA: South Australia, TAS: Tasmania, VIC: Victoria

^b^MGO: methylglyoxal

^c^DHA: dihydroxyacetone. NA: not applicable.

There were five honey samples with ≤10mg/kg MGO (Table 1 and S1 Table). Of these, one was from Murraylands SA (unspecified *Leptospermum* sp.), three were from Northern Rivers (multifloral *L*. *liversidgei*, *L*. *laevigatum* and *L*. *polygalifolium*), and one from the Hunter region in NSW (multifloral *L*. *laevigatum*). These samples also showed minimal quantifiable DHA (<61 mg/kg).

HMF levels were mostly low, with only seven samples above the acceptable level of >40 mg/kg. When ranked according to the year of collection, the highest HMF concentrations were found in the oldest honey samples (Table 2 and S1 Table) harvested from hives between 1998 and 2002, which was to be expected as HMF increases with age. HMF levels in the honey samples did not correlate with MGO or DHA concentrations.

**Table 2:** Hydroxymethylfurfural (HMF) concentrations in Australian *Leptospermum* honey collected between 1998 and 2007.

Year of collection	No. samples tested	HMF* (mg/kg)	
		Mean ± SD	Range
1998	1	143	NA
2002	7	49 ± 26	4–81
2003	3	21 ± 6	16–27
2004	5	18 ± 7	11–29
2005	43	14 ± 8	0–52
2006	18	12 ± 12	12–33
2007	2	17 ± 2	15–18

*HMF: hydroxymethylfurfural.

NA: not applicable.

### Non-peroxide activity (NPA) of Australian *Leptospermum* honey samples

The mean NPA of most of the samples (57 of the 80 samples) was above the previously defined [12] therapeutically beneficial level of >10% (Table 3 and S2 Table). Honey samples with high NPA between 15–20% (n = 29) were mostly monofloral *L*. *polygalifolium* and also included two *L*. *liversidgei*, one *L*. *laevigatum* and one *L*. *semibaccatum* sample, all of which were sourced from Northern Rivers. The 13 honeys with exceptionally high NPA (>20%) consisted mainly of *L*. *polygalifolium* (n = 9) from Northern Rivers (8 samples) and Byfield (one sample). One *L*. *liversidgei* and three *Leptospermum* sp. honeys also showed NPA >20%, and these were from Northern Rivers (n = 1), Stradbroke Island QLD (n = 1) and of unknown geographic origin (n = 2). There were 22 honey samples that were at least as active as the New Zealand manuka control (NPA 18.6%) (S2 Table).

**Table 3:** Non-peroxide antibacterial activity of Australian *Leptospermum* honeys.

*Leptospermum* species	Geographic region^a^	No. of samples	Mean NPA^b^ (%) ± SD
*L*. *continentale*	Central VIC	2	0 ± 0
*L*. *laevigatum*	Central VIC	1	0 ± 0
	Northern Rivers NSW	1	18.5 ± 0.2
*L*. *laevigatum* blend	Hunter NSW	1	0 ± 0
	Northern Rivers NSW	1	0 ± 0
*L*. *liversidgei*	Northern Rivers NSW	5	12.2 ± 8
*L*. *liversidgei* blend	Northern Rivers NSW	13	2.8 ± 5.4
*L*. *polygalifolium*	Byfield QLD	1	22.9 ± 4.4
	Northern Rivers NSW	28	18.9 ± 5.4
*L*. *polygalifolium*blend	Northern Rivers NSW	12	11.8 ± 6.2
*L*. *semibaccatum* blend	Northern Rivers NSW	4	14.8 ± 0.4
Unspecified *Leptospermum* sp.	Murraylands SA	1	0 ± 0
	Northern Rivers NSW	6	17 ± 3.3
	Stradbroke Island QLD	1	21.9 ± 1.2
	Unknown	3	15.3 ± 13.5

^a^NSW: New South Wales, QLD: Queensland, SA: South Australia, TAS: Tasmania, VIC: Victoria

^b^NPA: non-peroxide activity.

Honey samples that did not have detectable NPA (n = 20) were derived from a variety of *Leptospermum* species and from different regions, including monofloral *L*. *liversidgei*, a multifloral *L*. *liversidgei* sample, and a multifloral *L*. *laevigatum* sample from Northern Rivers and Hunter; monofloral *L*. *continentale* (Central VIC); and unspecified *Leptospermum* sp. from Murraylands. There were two samples of *L*. *polygalifolium* honey, one monofloral and one multifloral, that did not have detectable NPA and these were from Northern Rivers.

### Correlation between MGO and NPA in Australian *Leptospermum* honey

**Fig 1:**
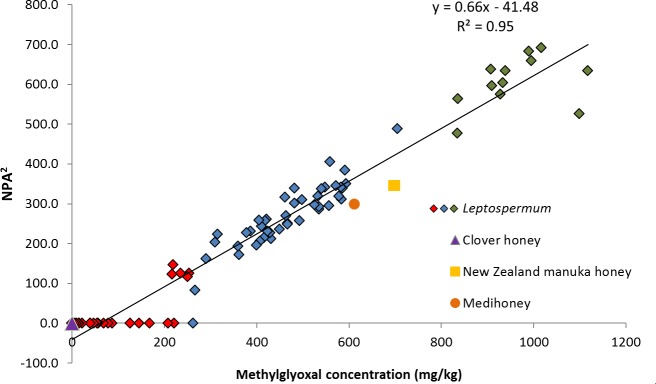
Relationship between methylglyoxal and non-peroxide activity in Australian *Leptospermum* honeys. Positive linear correlation between methylglyoxal (MGO) concentration and mean-squared non-peroxide activity (NPA) in Australian *Leptospermum* honey samples (n = 80) represented by diamond markers; green: MGO >800 mg/kg (NPA >20%), blue: MGO 260–800 mg/kg (NPA 10–20%), red: MGO <260 mg/kg (NPA <10%). Clover honey (purple triangle) served as a negative control with no detectable MGO or NPA, and New Zealand manuka honey (yellow square) and Medihoney^TM^ (orange circle) both served as the positive controls.

A strong positive linear correlation (r^2^ = 0.95, Pearson’s rho = 0.97, p<0.05) between MGO concentration and the mean-squared NPA for the Australian *Leptospermum* species honeys assessed in this study was observed, as shown in Fig 1. The mean-squared NPA was used to investigate this relationship following studies on New Zealand manuka honey [23–25]. Based on the New Zealand manuka relationship and data from the current study, an MGO concentration of >260 mg/kg was identified as giving NPA levels of >10% and samples with >800 mg/kg MGO were identified as having high NPA of >20%.

The 12 honeys with the highest MGO concentration (>800 mg/kg, coloured green in Fig 1) possessed the highest NPA ranging from 21.9–26.3% (Fig 1 and S2 Table). These samples were sourced from NSW (Northern Rivers) or QLD (Stradbroke Island and Byfield) and were either monofloral *L*. *polygalifolium* (n = 9) or other unknown *Leptospermum* sp. samples (n = 3).

Samples with MGO levels between 260–800 mg/kg (n = 40; coloured blue in Fig 1) had NPA values of 13.1–22.1%. These samples were primarily sourced from Northern Rivers NSW and most were monofloral *L*. *polygalifolium* honeys, although the highest MGO concentration (704 mg/kg, NPA 22.1%) in this range was found in a monofloral *L*. *liversidgei* honey from the same region.

Most samples that displayed detectable NPA (i.e. >9%) also possessed MGO above 260 mg/kg, with the exception of one *L*. *continentale* sample from Victoria, that had 262 mg/kg MGO but no detectable NPA. Honeys with 207–260 mg/kg MGO (coloured red in Fig 1) had NPA ranging from 0.0–12.1%. Three honey samples with MGO in this range did not have any detectable NPA; of these two were monofloral *L*. *continentale* from Victoria, and the other was a monofloral *L*. *liversidgei* honey from the Northern Rivers region of NSW. All of the remaining honeys with MGO content in this lower range that had NPA were *L*. *polygalifolium* samples (monofloral and multifloral) from the Northern Rivers region of NSW.

### Change in non-peroxide antibacterial activity of *Leptospermum* honey over time

**Fig 2:**
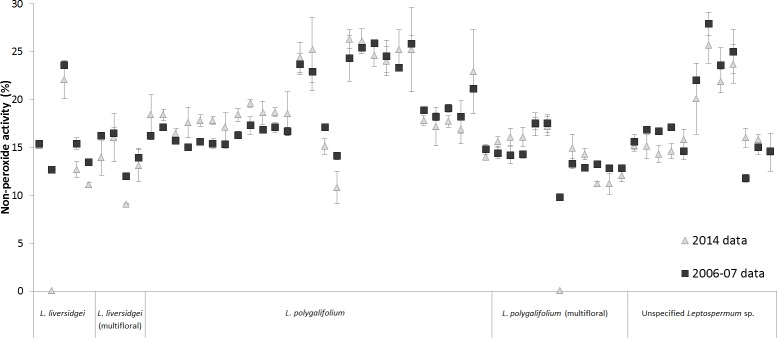
Non-peroxide activity (NPA) of Australian *Leptospermum* honey samples tested seven years apart. Australian *Leptospermum* honey samples (n = 61) identified by Irish *et al*. (2011) as having NPA. Dark grey boxes represent the NPA determined for the honeys at the time of collection (tested 2006–07). Light grey triangles represent NPA tested seven years post-collection in 2014, following storage of the samples in the dark at 4°C.

Irish *et al*. tested the 80 Australian *Leptospermum* honey samples at the time of collection (2006–2007) and found 61 had detectable NPA [12]. Repeat testing presented here found only two samples had completely lost activity following seven year storage at 4°C (Fig 2). These samples were a monofloral *L*. *liversidgei* honey (initial activity: 12.7%) and a multifloral *L*. *poligalifolium* honey (initial activity: 9.8%). The remaining samples showed little to no loss of NPA with <3% overall change in activity post storage for all but two samples. These two samples, one monofloral *L*. *polygalifolium* honey and one unspecified *Leptospermum* sp. honey, had a 3.3% and 4.2% decrease in NPA, respectively. The mean NPA of the *Leptospermum* honeys analysed by floral source was not significantly different to the initial NPA determined by Irish *et al*. (Wilcoxon signed ranks tests, p = 0.669), indicating that the NPA is stable for at least seven years under storage at 4°C in the dark. The majority of the *L*. *polygalifolium* samples (18 out of 27 monofloral, five out of 11 multifloral samples) had increased NPA relative to the initial values determined seven years prior, and this may have been due to the conversion of DHA to MGO in these samples over time.

## Discussion

Alternatives for treating infections are urgently needed due to the major global health threat posed by antibiotic resistance. The potent antibacterial activity of medical-grade manuka honey, derived from the New Zealand variety of *Leptospermum scoparium*, is now well established. The majority of its activity is attributed to its phytochemical constituents (in particular MGO derived from its precursor DHA). The use of manuka honey for the management and treatment of wounds could support current treatments and reduce the burden of antibiotic resistance in the wound sector. However, New Zealand is unlikely to be able to support the future growing demand for medical-grade honey.

Since the majority of the genus *Leptospermum* is endemic to and abundant in Australia, it is likely that there is an untapped source of medical-grade honey in Australia. The antimicrobial activity of Australian *Leptospermum* honey is assumed to be related to the same phytochemical components present in manuka honey, but this has never been tested. Therefore, the aim of this study was to survey a range of Australian *Leptospermum* honeys for NPA and simultaneously quantify the major phytochemical constituents contributing to this activity. Since the NPA of the collection of honeys used was previously determined [12], repeat testing also allowed us to assess the changes in NPA after seven years.

We have now performed the most comprehensive chemical assay of Australian *Leptospermum* honeys to date. Here we report a strong correlation (r^2^ = 0.95) between MGO content and NPA in Australian *Leptospermum* honey. Honey samples with the highest MGO and NPA also had the highest DHA concentrations, and these were mostly derived from either *Leptospermum polygalifolium*, *L*. *liversidgei* or mixed *Leptospermum*. We show increases in HMF levels in the honey samples with age (consistent with previous reports [37]), and no correlation between HMF and antibacterial activity (NPA), nor with the other phytochemicals (MGO, DHA). Our data also demonstrate for the first time that the NPA is preserved for up to seven years after original harvest when honeys are stored in the dark at 4°C.

### Methylglyoxal content correlates with non-peroxide antibacterial activity in Australian *Leptospermum* honey

We demonstrate a positive linear relationship between MGO concentration and mean-squared NPA in Australian *Leptospermum* honey. This is the first report of this type of relationship, outside of New Zealand’s manuka honey [23, 38]. This indicates that like New Zealand manuka, the NPA of Australian *Leptospermum* honey is due primarily to MGO (derived from DHA), and is a suitable alternative supply of medical-grade honey for use in wound care.

A minimum MGO concentration of approximately 200 mg/kg was seen in these Australian *Leptospermum* honeys to produce a NPA of approximately 9% (Fig 1), which is the lowest detectable NPA in this assay. This minimum detectable NPA concentration identified in this survey is consistent with previous findings [23, 24, 38, 39]. Atrott and Henle [39] described a strong correlation between MGO and NPA in New Zealand manuka honeys within a MGO concentration of 200–800 mg/kg, which we also demonstrate. Although there are likely to be other constituents of *Leptospermum* honeys that contribute to antibacterial activity [40, 41, 42], the tight correlation between MGO and NPA we have identified can be used by Australian beekeepers to estimate the antibacterial, and subsequent commercial, value of their honey.

### Some Australian *Leptospermum* honeys have exceptionally high MGO and DHA concentrations

We show that 75 of the 80 *Leptospermum* honeys from the Irish *et al*. collection [12] possessed both MGO and DHA (Table 1 and S1 Table). Honeys that possessed high MGO (>800 mg/kg) were generally associated with high concentrations of DHA (>2000 mg/kg), as has been seen in New Zealand manuka honey [25, 40]. The high levels of DHA and MGO in some of the Australian *Leptospermum* honey samples in our study are comparable to or higher than those observed in New Zealand manuka honey [23, 24, 39].

Many of these Australian honeys with very high levels of DHA and MGO honeys were classified as monofloral *L*. *polygalifolium* honeys or multifloral *Leptospermum* sp. from Northern Rivers. MGO was also present in moderate concentrations (400–700 mg/kg) in monofloral and multifloral samples of *L*. *liversidgei* and unspecified *Leptospermum* sp. honeys sourced from the same region. This concurs with a recent study investigating the DHA content in Australian *Leptospermum* nectar samples from Northern Rivers, which also showed high DHA in the samples [26] and another study showing high DHA and MGO in *Leptospermum* honeys from the same region [27].

Most of the *Leptospermum* honeys from Irish *et al*. [12] study were from Northern Rivers, however identifying new regions of Australia that may have a supply of high MGO and DHA content honey (and therefore excellent antibacterial activity) would be of great value for apiarists seeking to capitalise on the production of medical-grade honeys and to wound care clinicians. Investigating further Australian *Leptospermum* honeys and nectars is warranted, in particular taking in more geographical regions and *Leptospermum* species. We are currently investigating these aspects of Australian honeys. Our results indicate that various Australian *Leptospermum* honeys have similar important attributes to the well-characterised New Zealand manuka honey, with significant promise in the wound care arena, particularly as an infection control agent.

### Storage of *Leptospermum* honey at low temperature preserves non-peroxide activity (NPA)

Honey with potent NPA is highly sought after for the production of medical-grade honey wound care products due to its potential clinical value in treating skin and wound infections. We found that there was no significant change in the mean non-peroxide activity of the *Leptospermum* honeys (Fig 1) after seven years, establishing for the first time that this activity is retained over a long period if stored away from light and at 4°C. This suggests that the constituents contributing to NPA are also stable under these storage conditions. The stability of NPA over an extended period of time has positive implications. For example, storage of medicinal honey products under these conditions can maintain the high levels of antibacterial activity and also extend the shelf life of such products, thereby reducing waste and enabling greater use.

Low temperature is also likely to have slowed or halted the conversion of DHA to MGO [27, 40, 42, 43]. This could have other implications in the storage of medical-grade honeys. For example, honeys fresh from the hive with a high DHA content should potentially be stored under a particular set of conditions to maximise the conversion of DHA to MGO, and then moved to storage at low temperatures (4°C) to halt the conversion and maintain a high MGO concentration. However, since the original analysis did not include chemical quantitation [12] further investigation is needed.

## Conclusions

The data reported here show that Australian *Leptospermum* honeys have potential for therapeutic use as antibacterial agents as they possess a stable, non-peroxide antibacterial activity derived primarily from their phytochemical constituents. As is the case with New Zealand manuka, NPA correlates strongly with the MGO concentration in the honey, and high levels of DHA were also detected in many of the honey samples suggesting that there is an untapped supply of antibacterial *Leptospermum* honey in Australia. As most of the *Leptospermum* genus is endemic to Australia, it is likely that there are more valuable sources for medical-grade honey, providing Australian apiarists the opportunity to benefit from the lucrative medicinal honey market. An increased availability of medical-grade honey, together with scientific data on how it acts to kill bacterial cells will encourage its use in mainstream medicine, helping to reduce some of the burden of the antibiotic resistance crisis, particularly in wound care.

## Supporting Information

S1 Fig Regions of Australia where *Leptospermum* honeys were sourced. Samples received from Queensland (QLD) regions: Byfield (orange) and Stradbroke Island (green); New South Wales (NSW) regions: Northern Rivers (blue) and Hunter (red); Victoria (VIC) region: Central (yellow) and South Australia (SA) region: Murraylands (purple).
(TIF)

S1 Table Methylglyoxal, dihydroxyacetone and hydroxymethylfurfural levels in each Australian *Leptospermum* honey sample. ^a^ NSW: New South Wales, QLD: Queensland, SA: South Australia, TAS: Tasmania, VIC: Victoria. Data represented as mean ± standard deviation, range and % relative standard deviation (% RSD) calculated from two separate assays.
(PDF)

S2 Table Non-peroxide activity and region of origin of each Australian *Leptospermum* honey sample tested at the time of collection and seven years post-collection. ^a^ NSW: New South Wales, QLD: Queensland, SA: South Australia, TAS: Tasmania, VIC: Victoria; ^b^ Non-peroxide activity tested at the time of collection (2007 data) and seven years after collection (2014) data. Data represented as the mean and standard deviation of duplicate assays tested on three separate occasions.
(PDF)
