# Supplementary figures and images for: The Antibacterial Activity of Australian Leptospermum Honey Correlates with Methylglyoxal Levels
Source: PLoS One. 2016 Dec 28;11(12):e0167780. doi: 10.1371/journal.pone.0167780 (PMC5193333; doi:10.1371/journal.pone.0167780)

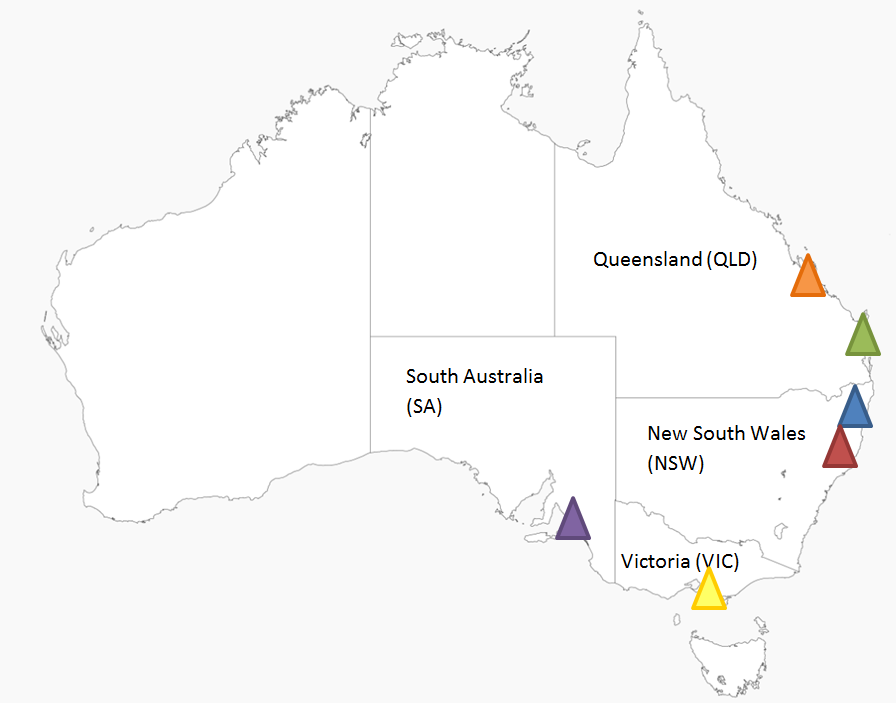

Supplement: S1 Fig — Samples received from Queensland (QLD) regions: Byfield (orange) and Stradbroke Island (green); New South Wales (NSW) regions: Northern Rivers (blue) and Hunter (red); Victoria (VIC) region: Central (yellow) and South Australia (SA) region: Murraylands (purple). (TIF) [file pone.0167780.s001.tif]
