# Supplementary material for: The Antibacterial Activity of Australian Leptospermum Honey Correlates with Methylglyoxal Levels
Source: PLoS One. 2016 Dec 28;11(12):e0167780. doi: 10.1371/journal.pone.0167780 (PMC5193333; doi:10.1371/journal.pone.0167780)
Supplement: S1 Table — a NSW: New South Wales, QLD: Queensland, SA: South Australia, TAS: Tasmania, VIC: Victoria. Data represented as mean ± standard deviation, range and % relative standard deviation (% RSD) calculated from two separate assays. (PDF) [file pone.0167780.s002.pdf]

Table S1. Methylglyoxal, dihydroxyacetone and hydroxymethylfurfural levels in Australian *Leptospermum* honey samples

Sample #	*Leptospermum* spp.	Region[a]	Methylglyoxal (mg/kg)					Dihydroxyacetone (mg/kg)					Hydroxymethylfurfural (mg/kg)				
			Mean	±	Range	SD	% RSD	Mean	±	Range	SD	% RSD	Mean	±	Range	SD	% RSD
55	*L. continentale*	Central VIC	262	±	11	16	6	775	±	19	27	3	13	±	1	1	8
57	*L. continentale*	Central VIC	222	±	4	5	2	1092	±	63	89	8	6	±	0	0	0
56	*L. laevigatum*	Central VIC	14	±	0	0	3	0	±	0	0	0	11	±	0	0	4
250	*L. laevigatum*	Northern Rivers NSW	583	±	7	9	2	1485	±	24	33	2	15	±	1	1	9
251	*L. laevigatum* and unknown	Hunter NSW	10	±	3	5	47	0	±	0	0	0	4	±	1	1	21
201	*L. laevigatum*, *Melaleuca nodosa*	Northern Rivers NSW	5	±	1	1	20	12	±	12	17	141	16	±	2	2	14
213	*L. liversidgei*	Northern Rivers NSW	704	±	7	10	1	882	±	3	5	1	21	±	1	1	3
151	*L. liversidgei*	Northern Rivers NSW	387	±	16	23	6	599	±	24	33	6	32	±	1	1	2
214	*L. liversidgei*	Northern Rivers NSW	291	±	1	1	0	304	±	1	2	1	16	±	2	3	20
215	*L. liversidgei*	Northern Rivers NSW	217	±	6	8	4	226	±	16	22	10	27	±	0	1	2
152	*L. liversidgei*	Northern Rivers NSW	208	±	17	24	12	257	±	20	29	11	29	±	2	2	8
211	*L. liversidgei, Aegicerus corniculatum*	Northern Rivers NSW	359	±	34	34	10	176	±	19	22	12	70	±	24	25	36
192	*L. liversidgei, Eucalyptus intermedia*	Northern Rivers NSW	362	±	18	25	7	818	±	48	67	8	15	±	1	2	13
188	*L. liversidgei, Eucalyptus intermedia*	Northern Rivers NSW	266	±	17	25	9	671	±	15	21	3	12	±	0	0	2
126	*L. liversidgei*/ *Eucalyptus* spp.	Northern Rivers NSW	88	±	5	7	8	297	±	1	2	1	8	±	1	1	14
142	*L. liversidgei*/ *Eucalyptus* spp.	Northern Rivers NSW	78	±	15	21	26	177	±	37	52	29	10	±	2	3	36
124	*L. liversidgei*/ *Eucalyptus* spp.	Northern Rivers NSW	69	±	6	9	12	199	±	4	6	3	8	±	1	1	14
122	*L. liversidgei*/ *Eucalyptus* spp.	Northern Rivers NSW	57	±	3	4	7	158	±	2	3	2	8	±	1	1	15
120	*L. liversidgei*/ *Eucalyptus* spp.	Northern Rivers NSW	55	±	3	4	7	178	±	2	3	2	6	±	0	0	6
123	*L. liversidgei*/ *Eucalyptus* spp.	Northern Rivers NSW	47	±	1	1	2	154	±	1	1	1	7	±	0	0	1
125	*L. liversidgei*/ *Eucalyptus* spp.	Northern Rivers NSW	41	±	2	3	7	141	±	3	5	3	5	±	1	1	15
127	*L. liversidgei*/ *Eucalyptus* spp.	Northern Rivers NSW	21	±	2	3	16	86	±	1	2	2	7	±	0	0	0
121	*L. liversidgei*/ *Eucalyptus* spp.	Northern Rivers NSW	16	±	1	1	5	78	±	3	4	6	6	±	0	0	2
119	*L. liversidgei*/ *Eucalyptus* spp.	Northern Rivers NSW	8	±	0	1	8	61	±	3	4	6	5	±	0	1	9

Sample #	*Leptospermum* spp.	Region[a]	Methylglyoxal (mg/kg)					Dihydroxyacetone (mg/kg)					Hydroxymethylfurfural (mg/kg)				
			Mean	±	Range	SD	% RSD	Mean	±	Range	SD	% RSD	Mean	±	Range	SD	% RSD
426	*L. polygalifolium*	Byfield QLD	1100	±	40	57	5	2182	±	106	150	6	20	±	0	0	1
290	*L. polygalifolium*	Northern Rivers NSW	1118	±	30	42	4	2371	±	106	150	6	20	±	0	0	1
281	*L. polygalifolium*	Northern Rivers NSW	1018	±	9	12	1	3313	±	29	42	1	14	±	2	3	19
282	*L. polygalifolium*	Northern Rivers NSW	989	±	13	18	2	3425	±	5	7	0	13	±	0	0	4
204	*L. polygalifolium*	Northern Rivers NSW	938	±	11	15	2	3272	±	15	22	1	11	±	1	1	10
283	*L. polygalifolium*	Northern Rivers NSW	934	±	4	5	1	3169	±	22	31	1	13	±	0	0	2
284	*L. polygalifolium*	Northern Rivers NSW	928	±	23	33	4	3188	±	4	6	0	16	±	0	1	4
203	*L. polygalifolium*	Northern Rivers NSW	910	±	13	18	2	3353	±	37	53	2	9	±	0	0	0
289	*L. polygalifolium*	Northern Rivers NSW	908	±	19	27	3	2829	±	23	32	1	11	±	1	1	7
140	*L. polygalifolium*	Northern Rivers NSW	593	±	2	2	0	2541	±	1	1	0	9	±	2	3	28
138	*L. polygalifolium*	Northern Rivers NSW	592	±	9	13	2	2450	±	14	20	1	9	±	0	0	4
130	*L. polygalifolium*	Northern Rivers NSW	585	±	10	15	2	2255	±	1	1	0	0	±	0	0	0
293	*L. polygalifolium*	Northern Rivers NSW	584	±	6	8	1	1150	±	25	36	3	14	±	0	0	3
291	*L. polygalifolium*	Northern Rivers NSW	579	±	1	2	0	1125	±	1	2	0	15	±	0	0	2
139	*L. polygalifolium*	Northern Rivers NSW	571	±	10	14	2	2392	±	25	35	1	10	±	2	3	26
292	*L. polygalifolium*	Northern Rivers NSW	558	±	23	32	6	1052	±	41	57	5	15	±	0	0	1
141	*L. polygalifolium*	Northern Rivers NSW	548	±	4	6	1	2047	±	3	4	0	9	±	0	0	3
137	*L. polygalifolium*	Northern Rivers NSW	541	±	1	1	0	2298	±	65	91	4	10	±	0	0	4
294	*L. polygalifolium*	Northern Rivers NSW	535	±	8	11	2	1004	±	23	33	3	14	±	0	0	0
136	*L. polygalifolium*	Northern Rivers NSW	534	±	6	8	1	2109	±	24	33	2	17	±	0	0	0
135	*L. polygalifolium*	Northern Rivers NSW	533	±	9	13	2	2217	±	92	130	6	10	±	1	2	19
132	*L. polygalifolium*	Northern Rivers NSW	499	±	5	7	1	2057	±	30	43	2	10	±	0	1	7
129	*L. polygalifolium*	Northern Rivers NSW	482	±	0	0	0	2070	±	7	10	0	10	±	1	2	17
131	*L. polygalifolium*	Northern Rivers NSW	464	±	3	4	1	1893	±	30	43	2	10	±	1	1	8
133	*L. polygalifolium*	Northern Rivers NSW	462	±	3	4	1	2005	±	44	62	3	9	±	0	1	6
511	*L. polygalifolium*	Northern Rivers NSW	400	±	0	0	0	762	±	4	5	1	18	±	1	1	8
216	*L. polygalifolium*	Northern Rivers NSW	379	±	2	3	1	324	±	10	14	4	45	±	1	2	4

Sample #	*Leptospermum* spp.	Region[a]	Methylglyoxal (mg/kg)					Dihydroxyacetone (mg/kg)					Hydroxymethylfurfural (mg/kg)				
			Mean	±	Range	SD	% RSD	Mean	±	Range	SD	% RSD	Mean	±	Range	SD	% RSD
217	*L. polygalifolium*	Northern Rivers NSW	251	±	3	4	1	157	±	6	8	5	81	±	0	0	0
128	*L. polygalifolium*	Northern Rivers NSW	146	±	5	7	4	351	±	1	1	0	12	±	0		0
145	*L. polygalifolium* and *L. speciosum*	Northern Rivers NSW	423	±	18	26	6	1262	±	3	5	0	15	±	1	2	14
144	*L. polygalifolium* and *L. speciosum*	Northern Rivers NSW	411	±	3	4	1	1241	±	9	13	1	11	±	0	0	4
206	*L. polygalifolium* and unknown	Northern Rivers NSW	526	±	4	6	1	914	±	12	17	2	17	±	0	1	3
205	*L. polygalifolium* and unknown	Northern Rivers NSW	482	±	27	38	8	835	±	22	32	4	15	±	0	0	2
512	*L. polygalifolium* and unknown	Northern Rivers NSW	169	±	3	4	2	370	±	8	11	3	15	±	1	1	6
149	*L. polygalifolium*, *Guioa semiglauca*	Northern Rivers NSW	315	±	0	0	0	918	±	13	19	2	18	±	0	0	0
187	*L. polygalifolium*, *Guioa semiglauca*	Northern Rivers NSW	311	±	9	13	4	826	±	35	50	6	14	±	2	3	19
219	*L. polygalifolium*, *L. whitei*	Northern Rivers NSW	253	±	17	25	10	216	±	16	23	11	4	±	0	0	0
218_A	*L. polygalifolium*, *L. whitei*	Northern Rivers NSW	236	±	8	11	5	238	±	8	11	5	43	±	1	2	4
218_B	*L. polygalifolium*, *L. whitei*	Northern Rivers NSW	218	±	7	10	5	209	±	9	13	6	37	±	1	1	2
207	*L. polygalifolium*, *Macadamia integrifolia*	Northern Rivers NSW	0	±	0	0	0	0	±	0	0	0	23	±	5	7	29
Manuka UTS	*L. scoparium* (New Zealand)	New Zealand	698	±	88	125	18	3852	±	540	569	15	6	±	2	2	33
286	*L. semibaccatum*, *Melaleuca nodosa*	Northern Rivers NSW	430	±	8	11	3	692	±	1	1	0	17	±	4	5	31
285	*L. semibaccatum*, *Melaleuca nodosa*	Northern Rivers NSW	424	±	1	2	0	736	±	11	15	2	15	±	1	1	6
288	*L. semibaccatum*, *Melaleuca nodosa*	Northern Rivers NSW	416	±	13	18	4	427	±	19	27	6	33	±	0	1	2
287	*L. semibaccatum*, *Melaleuca nodosa*	Northern Rivers NSW	407	±	8	12	3	657	±	2	3	0	14	±	1	1	6
495	*Leptospermum* sp.	Murraylands SA	0	±	0	0	0	0	±	0	0	0	14	±	0	0	1
49	*Leptospermum* sp.	Northern Rivers NSW	836	±	11	16	2	965	±	13	18	2	52	±	1	2	3
50	*Leptospermum* sp.	Northern Rivers NSW	493	±	63	69	14	1705	±	44	49	3	14	±	4	4	32
42	*Leptospermum* sp.	Northern Rivers NSW	468	±	27	30	6	1647	±	79	84	5	12	±	1	1	10
114	*Leptospermum* sp.	Northern Rivers NSW	468	±	7	10	2	1379	±	34	48	4	11	±	1	1	8
115	*Leptospermum* sp.	Northern Rivers NSW	432	±	10	15	3	1326	±	15	21	2	11	±	1	1	9
212	*Leptospermum* sp. and *Aegicerus corniculatum*	Northern Rivers NSW	418	±	1	2	0	206	±	2	3	2	65	±	6	8	12
47	*Leptospermum* sp.	Stradbroke Island QLD	835	±	25	36	4	2343	±	92	131	6	28	±	2	3	12
44	*Leptospermum* sp.	Unknown	995	±	10	9	1	501	±	26	24	5	160	±	55	60	37

Sample #	*Leptospermum* spp.	Region[a]	Methylglyoxal (mg/kg)					Dihydroxyacetone (mg/kg)					Hydroxymethylfurfural (mg/kg)				
			Mean	±	Range	SD	% RSD	Mean	±	Range	SD	% RSD	Mean	±	Range	SD	% RSD
43	*Leptospermum* sp.	Unknown	559	±	10	14	2	79	±	13	18	23	143	±	4	5	4
169	*Leptospermum* sp.	Unknown	126	±	3	4	3	186	±	10	14	8	33	±	1	1	3

[a] NSW: New South Wales, QLD: Queensland, SA: South Australia, TAS: Tasmania, VIC: Victoria.

Data represented as mean ± standard deviation, range and % relative standard deviation (% RSD) calculated from two separate assays.
