# Supplementary material for: The Antibacterial Activity of Australian Leptospermum Honey Correlates with Methylglyoxal Levels
Source: PLoS One. 2016 Dec 28;11(12):e0167780. doi: 10.1371/journal.pone.0167780 (PMC5193333; doi:10.1371/journal.pone.0167780)
Supplement: S2 Table — a NSW: New South Wales, QLD: Queensland, SA: South Australia, TAS: Tasmania, VIC: Victoria; b Non-peroxide activity tested at the time of collection (2007 data) and seven years after collection (2014) data. Data represented as the mean and standard deviation of duplicate assays tested on three separate occasions. (PDF) [file pone.0167780.s003.pdf]

Table S2. Non-peroxide activity and region of origin of Australian *Leptospermum* honey sample tested at the time of collection and seven years post-collection

Sample #	*Leptospermum* spp.	Region[a]	Non-peroxide activity[b] (%) Mean 2014	SD 2014	Mean 2007	SD 2007
55	*L. continentale*	Central VIC	0	0	0	0
57	*L. continentale*	Central VIC	0	0	0	0
56	*L. laevigatum*	Central VIC	0	0	0	0
250	*L. laevigatum*	Northern Rivers NSW	18.5	0.2	19.7	0.1
251	*L. laevigatum* and unknown	Hunter NSW	0	0	0	0
201	*L. laevigatum*, *Melaleuca nodosa*	Northern Rivers NSW	0	0	0	0
213	*L. liversidgei*	Northern Rivers NSW	22.1	2	23.6	0.4
151	*L. liversidgei*	Northern Rivers NSW	15.2	0	15.4	0.1
214	*L. liversidgei*	Northern Rivers NSW	12.7	0.8	15.4	0.6
215	*L. liversidgei*	Northern Rivers NSW	11.1	0.2	13.4	0.2
152	*L. liversidgei*	Northern Rivers NSW	0	0	12.7	0
211	*L. liversidgei, Aegicerus corniculatum*	Northern Rivers NSW	13.9	1.8	16.2	0.1
192	*L. liversidgei, Eucalyptus intermedia*	Northern Rivers NSW	13.1	1.7	13.9	1
188	*L. liversidgei, Eucalyptus intermedia*	Northern Rivers NSW	9.1	0.1	12	0
126	*L. liversidgei*/ *Eucalyptus* spp.	Northern Rivers NSW	0	0	0	0
142	*L. liversidgei*/ *Eucalyptus* spp.	Northern Rivers NSW	0	0	0	0
124	*L. liversidgei*/ *Eucalyptus* spp.	Northern Rivers NSW	0	0	0	0
122	*L. liversidgei*/ *Eucalyptus* spp.	Northern Rivers NSW	0	0	0	0
120	*L. liversidgei*/ *Eucalyptus* spp.	Northern Rivers NSW	0	0	0	0
123	*L. liversidgei*/ *Eucalyptus* spp.	Northern Rivers NSW	0	0	0	0
125	*L. liversidgei*/ *Eucalyptus* spp.	Northern Rivers NSW	0	0	0	0
127	*L. liversidgei*/ *Eucalyptus* spp.	Northern Rivers NSW	0	0	0	0
121	*L. liversidgei*/ *Eucalyptus* spp.	Northern Rivers NSW	0	0	0	0
119	*L. liversidgei*/ *Eucalyptus* spp.	Northern Rivers NSW	0	0	0	0
426	*L. polygalifolium*	Byfield QLD	22.9	4.4	21.1	0.1
290	*L. polygalifolium*	Northern Rivers NSW	25.2	4.4	25.8	0.9
281	*L. polygalifolium*	Northern Rivers NSW	26.3	1	24.3	2.4
282	*L. polygalifolium*	Northern Rivers NSW	26.1	1.3	25.4	0.6
204	*L. polygalifolium*	Northern Rivers NSW	25.2	3.4	22.9	2
283	*L. polygalifolium*	Northern Rivers NSW	24.6	1.1	25.9	0.2
284	*L. polygalifolium*	Northern Rivers NSW	24	1.5	24.5	1.7
203	*L. polygalifolium*	Northern Rivers NSW	24.4	1.6	23.7	1.1
289	*L. polygalifolium*	Northern Rivers NSW	25.2	2.1	23.3	0.2
140	*L. polygalifolium*	Northern Rivers NSW	18.7	0.5	17.1	0.5
138	*L. polygalifolium*	Northern Rivers NSW	19.6	0.4	17.3	0.9
130	*L. polygalifolium*	Northern Rivers NSW	18.4	0.6	17.1	0.2
293	*L. polygalifolium*	Northern Rivers NSW	17.7	0.6	19.1	0.4
291	*L. polygalifolium*	Northern Rivers NSW	17.8	0.5	18.9	0.1
139	*L. polygalifolium*	Northern Rivers NSW	18.6	1.2	16.8	0.1
292	*L. polygalifolium*	Northern Rivers NSW	17.2	2	18.2	0.4
141	*L. polygalifolium*	Northern Rivers NSW	18.5	2.3	16.7	0.4

Sample #	*Leptospermum* spp.	Region[a]	Non-peroxide activity[b] (%) Mean 2014	SD 2014	Mean 2007	SD 2007
137	*L. polygalifolium*	Northern Rivers NSW	18.4	0.7	16.3	0.4
294	*L. polygalifolium*	Northern Rivers NSW	16.9	1.5	18.2	1.7
136	*L. polygalifolium*	Northern Rivers NSW	17.1	1.5	15.3	0.4
135	*L. polygalifolium*	Northern Rivers NSW	17.8	0.4	15.4	0.5
132	*L. polygalifolium*	Northern Rivers NSW	17.6	1.6	15	0
129	*L. polygalifolium*	Northern Rivers NSW	18.4	2.1	16.2	0.4
131	*L. polygalifolium*	Northern Rivers NSW	16.4	0.6	15.7	0.2
133	*L. polygalifolium*	Northern Rivers NSW	17.8	0.6	15.6	0.1
511	*L. polygalifolium*	Northern Rivers NSW	14	0.3	14.8	0.5
216	*L. polygalifolium*	Northern Rivers NSW	15.1	0.8	17.1	0.1
217	*L. polygalifolium*	Northern Rivers NSW	10.8	1.7	14.1	0.4
128	*L. polygalifolium*	Northern Rivers NSW	0	0	0	0
145	*L. polygalifolium* and *L. speciosum*	Northern Rivers NSW	16.1	0.9	14.2	0.9
144	*L. polygalifolium* and *L. speciosum*	Northern Rivers NSW	15.6	0.5	14.4	0.6
206	*L. polygalifolium* and unknown	Northern Rivers NSW	17.2	1	17.5	0.9
205	*L. polygalifolium* and unknown	Northern Rivers NSW	17.4	1.2	17.5	0.6
512	*L. polygalifolium* and unknown	Northern Rivers NSW	0	0	9.8	0.2
149	*L. polygalifolium*, *Guioa semiglauca*	Northern Rivers NSW	14.9	1.5	13.3	0.5
187	*L. polygalifolium*, *Guioa semiglauca*	Northern Rivers NSW	14.3	0.6	12.9	0.3
219	*L. polygalifolium*, *L. whitei*	Northern Rivers NSW	11.2	0.3	13.2	0.3
218_A	*L. polygalifolium*, *L. whitei*	Northern Rivers NSW	11.2	1.1	12.8	0
218_B	*L. polygalifolium*, *L. whitei*	Northern Rivers NSW	12.1	0.7	12.8	0
207	*L. polygalifolium*, *Macadamia integrifolia*	Northern Rivers NSW	0	0	0	0
Manuka UTS	*L. scoparium* (New Zealand)	New Zealand	18.6	3	n/a	n/a
286	*L. semibaccatum*, *Melaleuca nodosa*	Northern Rivers NSW	15.1	1.3	16.8	0.3
285	*L. semibaccatum*, *Melaleuca nodosa*	Northern Rivers NSW	15.2	0.6	15.6	0.8
288	*L. semibaccatum*, *Melaleuca nodosa*	Northern Rivers NSW	14.6	0.8	17.1	0
287	*L. semibaccatum*, *Melaleuca nodosa*	Northern Rivers NSW	14.3	0.9	16.7	0.1
495	*Leptospermum* sp.	Murraylands SA	0	0	0	0
49	*Leptospermum* sp.	Northern Rivers NSW	23.7	2	25	2.3
50	*Leptospermum* sp.	Northern Rivers NSW	16.0	1	11.8	0.4
42	*Leptospermum* sp.	Northern Rivers NSW	15.8	1.1	14.6	0.9
114	*Leptospermum* sp.	Northern Rivers NSW	15.8	0.6	15	0.7
115	*Leptospermum* sp.	Northern Rivers NSW	14.5	2	14.6	0.2
212	*Leptospermum* sp. and *Aegicerus corniculatum*	Northern Rivers NSW	16	2.5	16.5	0.6
47	*Leptospermum* sp.	Stradbroke Island QLD	21.9	1.2	23.6	1.8
44	*Leptospermum* sp.	Unknown	25.7	1.9	27.9	1.2
43	*Leptospermum* sp.	Unknown	20.1	3.7	22	0.2
169	*Leptospermum* sp.	Unknown	0	0	0	0

[a] NSW: New South Wales, QLD: Queensland, SA: South Australia, TAS: Tasmania, VIC: Victoria; [b] Non-peroxide activity tested at the time of collection (2007 data) and seven years after collection (2014) data.

Data represented as the mean and standard deviation of duplicate assays tested on three separate occasions.
